# Atrial fibrillation before and after transcatheter aortic valve implantation: short- and long-term clinical implications

**DOI:** 10.2459/JCM.0000000000001553

**Published:** 2023-12-08

**Authors:** Salvatore Arrotti, Fabio Alfredo Sgura, Giulio Leo, Marco Vitolo, Daniel Monopoli, Nicola Forzati, Valerio Siena, Matteo Menozzi, Paolo Cataldo, Marco Stuani, Vernizia Morgante, Paolo Magnavacchi, Davide Gabbieri, Vincenzo Guiducci, Giorgio Benatti, Luigi Vignali, Rosario Rossi, Giuseppe Boriani

**Affiliations:** a Cardiology Division, Department of Biomedical, Metabolic and Neural Sciences, University of Modena and Reggio Emilia, Policlinico di Moden; b Clinical and Experimental Medicine PhD Program, University of Modena and Reggio Emilia; c Cardiology Division, Baggiovara Hospital; d Cardiac Surgery Division, Hesperia Hospital, Modena; e Interventional Cardiology Unit, USL-IRCCS, Reggio Emilia; f Cardiology Division, Parma University Hospital, Parma, Italy

**Keywords:** acute kidney injury, atrial fibrillation, hospitalization, major bleeding, transcatheter aortic valve implantation

## Abstract

**Background:**

Patients with atrial fibrillation (AF) undergoing transcatheter aortic valve implantation (TAVI) have been associated with worse short-term outcomes compared with patients in sinus rhythm but data on long-term outcomes are limited. The aim of our study was to evaluate the association between AF and short- and long-term outcomes in patients undergoing TAVI.

**Methods:**

We retrospectively evaluated patients undergoing TAVI between 2012 and 2022 in four tertiary centres. Two different analyses were conducted: (i) in-hospital and (ii) postdischarge analysis. First, we evaluated the association between preexisting AF and short-term outcomes according to VARC-3 criteria. Second, we analyzed the association between AF at discharge (defined as both preexisting and new-onset AF occurring after TAVI) and long-term outcomes at median follow-up of 3.2 years (i.e. all-cause death, hospitalization and major adverse cardiovascular events).

**Results:**

A total of 759 patients were initially categorized according to the presence of preexisting AF (241 vs. 518 patients). The preexisting AF group had a higher occurrence of acute kidney injury [odds ratio (OR) 1.65; 95%confidence interval ( CI) 1.15–2.38] and major bleeding (OR 1.86, 95% CI 1.06–3.27). Subsequently, the population was categorized according to the presence of AF at discharge. At the adjusted Cox regression analysis, AF was independently associated with an increased risk of all-cause death and cardiovascular hospitalization [adjusted hazard ratio (aHR) 1.42, 95% CI 1.09–1.86], all-cause death and all-cause hospitalization (aHR 1.38, 95% CI 1.06–1.78) and all-cause hospitalization (aHR 1.59, 95% CI 1.14.2.22).

**Conclusions:**

In a real-world cohort of patients undergoing TAVI, the presence of AF (preexisting and new-onset) was independently associated with both short- and long-term adverse outcomes.

## Introduction

Aortic valve stenosis (AS) is the most common acquired valvular heart disease in developed countries.^1^ Transcatheter aortic valve implantation (TAVI) has proven to be comparable or even better than surgery in all risk groups and not only in those considered unsuitable for surgical aortic valve replacement (SAVR) or older than 75 years and with multiple comorbidities.^2–4^

With the expansion of TAVI intervention, it is essential to evaluate which risk factors predict clinical outcomes.^5,6^ Despite its lower invasiveness compared with SAVR, TAVI is not even free from complications.^7^ Preexisting atrial fibrillation (AF) in patients undergoing TAVI is common, being reported in up to 51%, and new-onset AF is frequently detected during the peri-procedural period.^8^ For this reason, AF impact on the prognosis of patients undergoing TAVI is a remarkable topic.^8^

However, despite the frequency of AF after the TAVI procedure and its impact on the following management and clinical outcomes, real-world data are limited.

Accordingly, the aim of the present study is to evaluate the impact of AF in patients treated with TAVI on the main cardiovascular outcomes, both in the immediate postprocedural in-hospital course and in postdischarge long-term follow-up.

## Methods

### Study design and cohort

In this multicentre and observational study, we retrospectively evaluated 759 patients with severe AS undergoing TAVI in four tertiary centres of Italy (North-West area of the Emilia Romagna, a region of the Northern Italy) between 2012 and 2022: (i) University Hospital Modena, (ii) S. Agostino Estense Baggiovara, (iii) University Hospital Parma, and (iv) St. Maria Nuova Hospital of Reggio Emilia.

All patients with severe aortic stenosis, diagnosed by echocardiography following the recommendations of the European and American Societies of Echocardiography,^9^ were included in the present analysis. Both high-gradient aortic stenosis (mean gradient ≥ 40 mmHg, peak velocity ≥ 4.0 m/s and valve area ≤ 1 cm^2^ or ≤ 0.6 cm^2^/m^2^) and low-flow low-gradient aortic stenosis (mean gradient < 40 mmHg, valve area < 1 cm^2^, Svi ≤ 35 ml/m^2^) types were included.^2^ Prior to the intervention, sociodemographic parameters, echocardiographic data and comorbidities were collected. All the screening and preoperatory assessments included coronary angiography, transthoracic echocardiography, contrast-enhanced multislice computer tomography scan of the chest and abdomen to assess aortic, iliac and femoral vessels with the evaluation of calcium amount.^10^

Eligibility for TAVI was assessed by the local Heart Team involving cardiac surgeons, interventional and clinical cardiologists and echocardiographers. Patients were classified according to the Society of Thoracic Surgeons (STS) score (high risk >8%, intermediate risk 4–8% and low risk <4%) and evaluated for percutaneous intervention according to current international recommendations.^2^ Motivations to refuse SAVR were the presence of contra-indications to cardiac surgery, e.g. porcelain aorta, frailty, and previous radiation therapy of the chest. The access site was evaluated based on the measurements of preprocedural multislice computed tomography scan. Valve implantation was performed via either a transfemoral (percutaneous or surgical) or a nontransfemoral approach (transaxillary or direct aortic). No transapical approaches were performed in our cohort. The prosthetic valve sizing and model (balloon expandable vs. self-expandable) were selected according to operators’ choice and patients’ characteristics. Clinical, echocardiographic, and procedural data were recorded by individual centres in a single, anonymized database.

### Study outcomes

In order to evaluate the impact of AF in patients with TAVI on clinical outcomes, two different analyses were conducted: (i) ‘in-hospital’ and (ii)‘postdischarge’ analysis.

The in-hospital analysis considered patients categorized into two groups: (i) ‘no history of AF’ and (ii) ‘preexisting AF’ defined as the presence of clinical AF independently of the pattern (i.e. paroxysmal, persistent or permanent).^11,12^ We evaluated the association between preexisting AF and short-term in-hospital outcomes, defined according to the Valve Academic Research Consortium-3 (VARC-3) consensus document.^13^ The composite of VARC-3 short-term outcome [acute kidney injury (AKI), stroke/TIA, minor bleeding, major bleeding, major vascular complication, minor vascular complication, need to pacemaker (PM) implantation, new left bundle branch block, new right bundle branch block, in-hospital death and cardiac tamponade] was considered as the primary in-hospital end point. The single components of the primary in-hospital composite outcome were considered as secondary in-hospital end points.

In the postdischarge analysis, we compared patients without history of AF vs clinical AF at the end of hospitalization, defined as both preexisting and new-onset AF that occurred after TAVI.^14^ For this second analysis, the composite of all-cause death and cardiovascular (CV) hospitalization in the long term was the primary postdischarge end point. Secondary postdischarge long-term outcomes were all-cause death, all-cause hospitalization, and major adverse cardiovascular events (MACE), defined as the composite of stroke/TIA, myocardial infarction, and CV death.

### Statistical analysis

Continuous variables were expressed as mean ± standard deviation (SD) and compared using analysis of variance (one-way ANOVA). Categorical variables were reported as counts and percentages and compared using Pearson chi-square or Fisher's exact test when appropriate.

For the in-hospital analysis, we used unadjusted logistic regression to establish the relationship between preexisting AF and individual VARC-3 outcomes (AKI, stroke/TIA, minor bleeding, major bleeding, major vascular complication, minor vascular complication, all bleeding, need to PM implantation, new left bundle branch block, new right bundle branch block, in-hospital death, and cardiac tamponade). Furthermore, we built an adjusted logistic regression model to evaluate the association between preexisting AF and the primary in-hospital composite outcome of VARC-3 and the secondary outcomes of AKI and major bleeding. We focused on these two individual end points given their known clinical implications in AF patients undergoing TAVI. Age, sex, arterial hypertension, diabetes mellitus, renal function (evaluated according to CKD-EPI), previous stroke, previous coronary artery disease and left ventricular ejection fraction (LVEF) were used as adjustments. Results were expressed as odds ratio (OR) and 95% confidence interval (CI)

For the postdischarge analysis, we explored the relationship between AF at discharge (new onset or history of AF) and long-term outcomes. Plots of Kaplan–Meier curves for the composite primary postdischarge end point of all-cause death and CV hospitalization and secondary postdischarge end points (all-cause death, all-cause hospitalization, and MACE) according to AF at discharge were performed. Survival distributions were compared using the Log-Rank test.

Unadjusted and adjusted Cox regression analyses were performed for the postdischarge long-term outcomes. The same adjusted regression model as described above was used. The hazard ratio (HR) and the corresponding 95% CI, were reported.

All statistical analyses were performed using IBM SPSS Statistics 26.0 (IBM Corp., Armonk, NY).

## Results

A total of 759 patients were included, equally distributed by gender (402 females, 50.3%) with a mean age of 82.2 ± 5.2 years. Two different analyses were conducted: (i) ‘in-hospital’ and (ii)‘postdischarge’ analysis.

### In-hospital analysis

In the in-hospital analysis, we considered a total of 759 patients admitted for a TAVI procedure and stratified according to history of AF (‘no AF history’ = 518, 68.2% vs. ‘preexisting AF’ = 241, 31.8%). Baseline characteristics of the two groups are summarized in Table 1. In the preexisting AF group, patients showed higher rates of previous stroke, prior PM implantation and moderate/severe mitral regurgitation (Table 1). No differences were observed among groups regarding age, main CV risk factors (including hypertension, diabetes mellitus, smoking habit), access site, device type and amount of contrast medium used during the TAVI procedure (Table 1).

**Table 1:** In-hospital analysis. baseline characteristics of the cohort stratified by ‘no AF history’ until TAVI procedure vs. ‘preexisting AF’

		No AF history n = 518 (68.2%)	Preexisting AF n = 241 (31.8%)	*P*-value
Age and anthropometric factors				
Age	Years ± SD	82.3 ± 5.3	82.1 ± 5.1	0.63
Sex	F %	55.0 (*n* = 285)	48.5 (*n* = 117)	0.22
BMI	*n*	26.5 ± 4.6	26.7 ± 4.9	0.98
CV risk factors and comorbidity				
Diabetes mellitus	%	26.3 (*n* = 136)	25.3 (*n* = 61)	0.78
Dyslipidemia	%	68.7 (*n* = 356)	66.0 (*n* = 159)	0.24
Hypertension	%	89.6 (*n* = 464)	91.3 (*n* = 220)	0.46
CHA2DS2VASC score	Mean ± SD	4.36 ± 0.9	4.36 ± 1.0	0.34
Chronic pulmonary disease	%	16.8 (*n* = 87)	17.8 (*n* = 43)	0.80
Cancer history	%	12.4 (*n* = 64)	11.7 (*n* = 28)	0.78
Prior Stroke	%	8.5 (*n* = 44)	14.9 (*n* = 36)	**0.007**
Prior myocardial infarction	%	16.2 (*n* = 84)	14.5 (*n* = 35)	0.55
Coronary artery disease	%	33.8 (*n* = 175)	29.2 (*n* = 70)	0.20
Prior CABG	%	10.8 (*n* = 56)	8.7 (*n* = 21)	0.37
Prior PCI	%	24.1 (*n* = 125)	20.7 (*n* = 50)	0.30
Prior BAV	%	7.7 (*n* = 40)	7.1 (*n* = 17)	0.74
Prior SAVR	%	4.2 (*n* = 22)	5.8 (*n* = 14)	0.34
eGFR (CKD-EPI)	ml/min ± SD	57.9 ± 22	55.7 ± 25	0.37
CKD-eGFR CKD-EPI < 60 ml/min	%	59.1 (*n* = 306)	62.7 (*n* = 151)	0.34
Electrocardiographic characteristics				
Right bundle brunch block	%	6.8 (*n* = 35)	6.2 (*n* = 15)	0.78
Left bundle branch block	%	6.6 (*n* = 34)	8.3 (*n* = 20)	0.39
Prior PM implantation	%	6.0 (*n* = 31)	17.0 (*n* = 41)	**<0.001**
Echocardiographic parameters				
Ejection fraction	%	51.3 ± 9.5	49.6 ± 10.4	0.33
Moderate or severe AR	%	22.6 (*n* = 117)	18.7 (*n* = 45)	0.22
Moderate or severe MR	%	24.3 (*n* = 126)	37.3 (*n* = 90)	**0.001**
Procedural characteristics				
Access type (transfemoral)	%	97.9 (*n* = 507)	99.2 (*n* = 239)	0.20
Valve type	%			0.16
	S. expandable	54.2 (*n* = 281)	59.8 (*n* = 144)	
	B. expandable	45.8 (*n* = 237)	40.2 (*n* = 97)	
STS score	%			
	High	7.7 (*n* = 40)	7.1 (*n* = 17)	0.74
	Intermediate	34.0 (*n* = 176)	39.8 (*n* = 96)	0.12
	Low	58.3 (*n* = 302)	52.7 (*n* = 127)	0.15
Amount of contrast	Ml ± SD	214.1 ± 90	205.4 ± 89	0.72

Values are presented as *n* (%) or mean ± standard deviation. BMI, body mass index; CHA2DS2VASC, congestive heart failure or left ejection fraction ≤40%, hypertension, age ≥ 75 years, diabetes mellitus, prior stroke or TIA, vascular disease (including myocardial infarction and peripheral artery disease), age >65 years, female gender; AR, aortic regurgitation; BAV, balloon aortic valvuloplasty; CABG, coronary artery bypass graft; CKD, chronic kidney disease eGFR, estimated glomerular filtration rate; MR, mitral regurgitation; PCI, percutaneous coronary intervention; PM, pacemaker; SAVR, surgical aortic valve replacement; STS, Society of Thoracic Surgeons risk.

Table 2 shows the crude rates and the unadjusted OR with the corresponding 95% CI for the in-hospital adverse events according to the history of AF. Overall, 447 (58.8%) patients experienced at least one event of primary in-hospital composite outcome (VARC-3) without significant differences between the two groups. Considering the individual components of the primary in-hospital outcome, patients with a history of AF showed significantly higher rates of AKI and major bleeding. There were no statistically significant differences between the two groups regarding the occurrence of in-hospital death, stroke/TIA, bleedings, vascular complications, PM implantation, new-onset bundle branch blocks, and cardiac tamponade (Table 2).

**Table 2:** VARC-3 outcomes in ‘no AF history’ and ‘preexisting AF’ patients after TAVI

	No AF history	Preexisting AF	*P*-value	OR (95% CI)
In-hospital outcomes				
**Any VARC-3**	**60.4 (*n* = 313)**	**55.6 (*n* = 134)**	**0.21**	**0.84 (0.61–1.14)**
AKI	**7.3 (*n* = 38)**	**12.9 (*n* = 31)**	**<0.01**	**1.65 (1.15–2.38)**
Stroke/TIA	2.1 (*n* = 11)	1.7 *(*n = 4)	0.67	0.77 (0.24–2.46)
Minor bleeding	10.2 (*n* = 53)	7.5 (*n* = 18)	0.22	0.70 (0.40–1.23)
Major bleeding	**5.6 (*n* = 29)**	**10 (*n* = 24)**	**0.03**	**1.86 (1.06–3.27)**
Minor vascular complication	11.4 (*n* = 59)	10.8 (*n* = 26)	0.81	0.98 (0.60–1.59)
Major vascular complication	5.0 (*n* = 29)	7.5 (*n* = 24)	0.18	1.52 (0.82–2.84)
Need to PM implantation	15.6 (*n* = 81)	16.2 (*n* = 39)	0.85	1.04 (0.68–1.58)
New left bundle branch block	32.6 (*n* = 169)	27.8 (*n* = 67)	0.18	0.79 (0.56–1.11)
New right bundle branch block	1.4 (*n* = 7)	0.4 (*n* = 1)	0.24	0.30 (0.03–2.53)
In-hospital death	1.0 (*n* = 5)	1.7 (*n* = 4)	0.42	1.60 (0.35–7.24)
Cardiac tamponade	1.4 (*n* = 7)	0.8 (*n* = 2)	0.54	0.61 (0.12–2.98)

Values are presented as *n* (%). AKI, acute kidney injury; PM, pacemaker; TIA, transient ischemic attack; VARC-3, Valve Academic Research Consortium-3.

At the adjusted logistic regression analysis, preexisting AF was independently associated with a higher occurrence of AKI [adjusted OR (aOR) 1.65, 95% CI 1.13–2.41] and a trend toward a higher occurrence of major bleeding (aOR 1.77, 95% CI 0.99–3.16) (Table 3).

**Table 3:** Unadjusted and adjusted logistic regression analysis for the primary and secondary in-hospital outcomes

	Unadjusted analysis			Adjusted analysis^a^		
Primary in-hospital outcome	OR	95% CI	*P*	aOR	95% CI	*P*
Any VARC						
Preexisting AF (vs. *no AF history*)	0.84	0.61–1.14	0.26	0.86	0.62–1.18	0.35

Secondary in-hospital outcomes	OR	95% CI	*P*	aOR	95% CI	*P*
AKI						
Preexisting AF (vs. *no AF history*)	1.65	1.15–2.38	<0.01	1.65	1.13–2.41	<0.01
Major bleeding						
Preexisting AF (vs. *no AF history*)	1.86	1.06–3.27	0.03	1.77	0.99–1.13	0.05

CI, confidence interval; OR, odds ratio.

a Adjusted analysis for age, sex, arterial hypertension, diabetes mellitus, renal function (evaluated according to CKD-EPI), previous stroke, previous coronary artery disease and left ventricular ejection fraction.

### Postdischarge analysis

Nine patients died during the index hospitalization and a total of 750 patients were considered for the postdischarge analysis (397 females, 52.9%). As shown in Table 4, a total of 275 (36.6%) patients had a diagnosis of AF at discharge, including preexisting AF (241 patients) and new-onset AF (34 patients).

**Table 4:** Postdischarge analysis. Baseline characteristics of the cohort stratified by ‘no AF at discharge’ vs. ‘AF at discharge’ (both preexisting and new-onset AF)

		No AF at discharge *n* = 475 (63.3%)	AF at discharge *n* = 275 (36.7%)	*P*-value
Age and anthropometric factors				
Age	Years ± SD	82.3 ± 5.4	82.2 ± 5.0	0.40
Sex	F %	54.1 (*n* = 257)	50.9 (*n* = 140)	0.40
BMI	N	26.4 ± 4.6	26.9 ± 4.9	0.52
CV risk factors and comorbidity				
Diabetes mellitus	%	26.9 (*n* = 128)	22.5 (*n* = 62)	0.18
Dyslipidemia	%	68.0 (*n* = 323)	66.9 (*n* = 184)	0.76
Hypertension	%	88.8 (*n* = 422)	92.0 (*n* = 253)	0.17
CHA2DS2VASc score	N	4.34 ± 0.9	4.38 ± 1.0	0.23
Chronic pulmonary disease	%	16.6 (*n* = 79)	18.9 (*n* = 52)	0.43
Cancer history	%	12.4 (*n* = 59)	12.0 (*n* = 33)	0.87
Prior stroke	%	8.2 (*n* = 39)	14.2 (*n* = 39)	**0.01**
Prior myocardial infarction	%	16.2 (*n* = 77)	14.2 (*n* = 39)	0.46
Coronary artery disease	%	33.5 (*n* = 159)	30.2 (*n* = 83)	0.35
Prior CABG	%	10.9 (*n* = 52)	9.1 (*n* = 25)	0.42
Prior PCI	%	24.0 (*n* = 114)	21.5 (*n* = 59)	0.43
Prior BAV	%	7.6 (*n* = 36)	7.3 (*n* = 20)	0.88
Prior SAVR	%	4.4 (*n* = 21)	5.5 (*n* = 15)	0.52
eGFR (CKD-EPI)	ml/min	57.4 ± 22	56.9 ± 24	0.43
Electrocardiographic characteristics				
Right bundle brunch block	%	6.7 (*n* = 32)	6.9 (*n* = 19)	0.93
Left bundle branch block	%	6.5 (*n* = 31)	8.4 (*n* = 23)	0.35
Prior PM implantation	%	5.5 (*n* = 26)	16.0 (*n* = 44)	**<0.001**
Echocardiographic parameters				
Ejection fraction	%	51.3 ± 9.6	50.0 ± 10.1	0.80
Moderate or severe AR	%	22.5 (*n* = 107)	18.9 (*n* = 52)	0.24
Moderate or severe MR	%	24.2 (*n* = 115)	35.6 (*n* = 98)	**0.001**
Procedural characteristics				
Access type	%			
	Transfemoral	99.4 (*n* = 472)	100 (*n* = 275)	0.19
Valve type	%			0.62
	S. expandable	55.8 (*n* = 268)	56.6 (*n* = 158)	
	B. expandable	44.4 (*n* = 211)	42.5 (*n* = 117)	
STS score	%			
	High	7.2 (*n* = 34)	6.9 (*n* = 19)	0.90
	Intermediate	34.3 (*n* = 163)	39.3 (*n* = 108)	0.17
	Low	58.5 (*n* = 278)	53.5 (*n* = 147)	0.18

Values are presented as *n* (%) or mean ± standard deviation. AR, aortic regurgitation; BAV, balloon aortic valvuloplasty; BMI, body mass index; CABG, coronary artery bypass graft; CHA2DS2VASC, congestive heart failure or left ejection fraction ≤40%, hypertension, age≥75 years, diabetes mellitus, prior stroke or TIA, vascular disease (including myocardial infarction and peripheral artery disease), age >65 years, female gender; CKD, chronic kidney disease eGFR, estimated glomerular filtration rate; MR, mitral regurgitation; PCI, percutaneous coronary intervention; PM, pacemaker; SAVR, surgical aortic valve replacement; STS, Society of Thoracic Surgeons risk.

Table 5 shows the comparison of the crude rates of major adverse events among the two groups. The median follow-up period was 3.2 years (range: from 1 to 5 years).

**Table 5:** Major adverse events during follow-up according to AF at discharge

		No AF at discharge *N* = 475	AF at discharge *N* = 275	*P*-value
All-cause death and CV hospitalization	%	27.2 (*n* = 129)	35.6 (*n* = 98)	<0.01
All-cause death and all-cause hospitalization	%	29.9 (*n* = 142)	38.2 (*n* = 105)	<0.01
All-cause death	%	18.1 (*n* = 86)	22.9 (*n* = 63)	0.05
All-cause hospitalization	%	15.8 (*n* = 75)	24.7 (*n* = 68)	<0.01
MACE (stroke, MI, CV death)	%	17.7 (*n* = 84)	21.1 (*n* = 58)	0.21

Values are presented as *n* (%). CV, cardiovascular; MACE, major adverse cardiac events; MI, myocardial infarction.

Overall, 227 (30.3%) patients experienced all-cause death or hospital readmissions for CV reasons. The occurrence of the composite outcomes of all-cause death/CV hospitalization and all-cause death/all-cause hospitalization was higher in the AF patients (35.6% and 38.2% respectively). These findings were consistent also when considering all-cause death and hospitalization alone (Table 5). There were no significant differences regarding the composite outcome of MACE (Table 5).

**Fig. 1:**
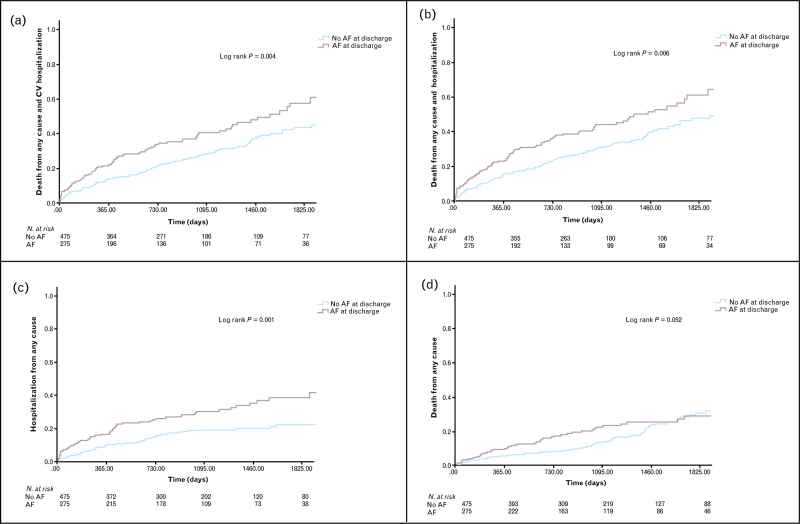
Kaplan–Meier curves for the primary and secondary long-term outcomes according to the presence of AF at discharge.

Kaplan–Meier curves for the primary and secondary postdischarge end points are shown in Fig. 1.

The unadjusted and adjusted Cox regression analyses estimating the association between AF at discharge and long-term clinical outcomes are reported in Table 6.

**Table 6:** Unadjusted and adjusted Cox regression analysis for the primary and secondary postdischarge long-term outcomes

	Unadjusted analysis			Adjusted analysis^a^		
Primary postdischarge outcome	HR	95% CI	*P*	aHR	95% CI	*P*
All-cause death and CV hospitalization						
*AF at discharge (vs. no AF)*	1.47	1.13–1.91	<0.01	1.42	1.09–1.86	0.01

Secondary postdischarge outcomes	HR	95% CI	p	aHR	95% CI	p
*AF at discharge (vs. no AF)*						
All-cause death and all-cause hospitalization	1.42	1.10–1.83	<0.01	1.38	1.06–1.78	0.02
All-cause death	1.38	1.00–1.93	0.05	1.30	0.94–1.80	0.10
All-cause hospitalization	1.70	1.23–2.37	<0.01	1.59	1.14–2.22	<0.01
MACE (stroke, IMA, CV death)	1.24	0.89–1.74	0.21	1.16	0.83–1.62	0.37

CI, confidence interval; HR, hazard ratio.

a Adjusted analysis for age, sex, arterial hypertension, diabetes mellitus, renal function (evaluated according to CKD-EPI), previous stroke, previous coronary artery disease and ejection fraction.

At the unadjusted Cox regression analysis, AF at discharge was associated with a higher risk of all-cause death and CV hospitalization (HR 1.47, 95% CI 1.13–1.91), all-cause death and all-cause hospitalization (HR 1.42, 95% CI 1.10–1.83) and hospitalization alone (HR 1.70, 95% CI 1.23–1.37). The adjusted Cox regression analysis showed consistent results: AF at discharge was independently associated with an increased risk of all-cause death and CV hospitalization [adjusted HR (aHR) 1.42, 95% CI 1.09–1.86], all-cause death and all-cause hospitalization (aHR 1.38, 95% CI 1.06–1.78) and hospitalization alone (aHR 1.59, 95% CI 1.14–2.22). No statistically significant association was observed between AF at discharge and MACE, while there was a nonsignificant trend towards a higher risk of all-cause death (aHR 1.30, 95% CI 0.94–1.80) (Table 6).

## Discussion

The main findings of our study are as follows: (i) patients with AF undergoing TAVI are more likely to develop AKI and to experience major bleeding; (ii) preexisting AF and new-onset AF both were associated with a higher risk of the composite outcome of all-cause death and CV hospitalization, as well as the composite outcome of all-cause death and all-cause hospitalization and all-cause hospitalization alone in the long term.

Approximately one-third of patients treated with TAVI regularly experience AKI, which is linked to a longer hospital stay and a higher mortality rate.^15^

The TAVI procedure includes the administration of contrast medium (CM) and episodes of pronounced hypotension (‘rapid pacing’) during balloon valvuloplasty and valve implantation, both these factors, added to patient's risk factors, increased the risk of AKI.^16^ In our cohort, the mean amount of contrast used was slightly lower in patients with AF. Despite this, the occurrence of AKI was higher in AF patients, highlighting that this risk is multifactorial, and the amount of contrast used is probably only one of the concomitant factors involved in the complex interplay between AKI and AF. The use of large-lumen aortic TAVI catheters increases the risk of renal cholesterol embolism, which may be also a risk factor for AKI due to the high frequency of concomitant generalized atherosclerosis in these patients.^17^

AKI is a strong predictor of 30-day all-cause mortality; particularly, AKI superimposed to severe chronic kidney disease (CKD).^18–20^

Our data were in accordance with those of Patil *et al.*, who examined retrospectively a cohort of 7266 patient who underwent TAVI between 2012 and 2015. AF was recorded in 44% of patients, showing association with AKI (OR 1.54, 95% CI 1.33, 1.78, *P* < 0.01) and blood transfusion (OR 1.14, 95% CI 1.00, 1.30, *P* = 0.05) but not with inpatient mortality (OR 1.09, 95% CI 0.81, 1.48, *P* = 0.57).^21^ More recently, Nso *et al.* performed a meta-analysis for a total of 15 studies that enrolled 158 220 patients who underwent TAVI. Primary clinical outcomes were 30-day mortality, stroke, early bleeding, and late bleeding; the secondary outcomes were AKI and length of stay. The authors found that atrial fibrillation was associated with higher risk of all primary and secondary outcomes. Specifically, patients with pre-AF compared with patients with sinus rhythm had a higher risk of AKI (OR 2.43, 95% CI 1.10–5.35, *P* = 0.03). New-onset AF, but not pre-AF, was associated with higher risk of 30-day mortality, stroke, and extended length of stay after TAVI.^22^

In relation to bleeding complications, we used the VARC-3 definition of complications that ensured a major homogeneity and comparability of data from different research centres.^13^

Wang *et al.* used the VARC definitions too and found that the patients with post-TAVI bleeding had an increased 30-day postoperative mortality compared with controls. Preexisting AF was correlated independently with TAVI-associated bleeding (OR 2.63, 95% CI 1.33–5.21, *P* = 0.005), likely due to AF-related anticoagulation.^23^

Also Angelillis *et al.* evaluated some clinical outcomes shared with VARC-3, in order to predict the length of the discharge after the TAVI procedure. High STS score, general anesthesia, high NYHA class and in-hospital onset of conduction disturbances and new PM implantation were associated with slower discharge.^24^

A slightly different analysis was made by Monosilio *et al.* and their evaluation included VARC outcomes, although they focused on postoperatory SIRS which resulted as a predictor of 24-month mortality.^25^

Patients with pre-AF had a higher probability of an early bleeding after TAVI than patients in sinus rhythm (OR 17.41, 95% CI 6.49–46.68, *P* = 0.03).^22^

Studies on mortality in patients with preexisting and new-onset AF after TAVI showed counteracting results.^13–15,17,18,21–23,26^ In 2013, Sortecki *et al.* carried out their study from a prospective registry including 389 high-risk patients undergoing TAVI. After 1 year, all-cause mortality was higher among patients with AF (30.9%) compared with those without AF (13.9%); (HR 2.36, 95% CI 1.43–3.90). This was observed irrespective of the type of AF (permanent, HR 2.47, 95% CI 1.40–4.38; persistent, HR 3.60, 95% CI 1.10–11.78; paroxysmal, HR 2.88, 95% CI 1.37–6.05). Mortality gradually increased with higher CHA2DS2–VASC scores (score 1–3: HR 2.20, 95% CI 0.92–5.27; score 6–8: HR 4.12, 95% CI 2.07–8.20). The risks of stroke and life-threatening bleeding were similar among patients with and without AF.^27^ According to different studies, not only preexisting AF was linked to a higher rate of late mortality,^14^ but also that new-onset AF is correlated with an increased risk of mortality.^27^ Furthermore, a new recent study showed an increase in early and late mortality in patients with preexisting and new-onset AF treated with TAVI.^12^

Ryan *et al.* in a meta-analysis of 179 studies showed that new-onset atrial fibrillation was associated with a near doubling of 30-day or in-hospital mortality after TAVI (RR 1.76, 95% CI 1.12–2.76).^28^ The relationship between increased mortality and AF is not clear but surely multifactorial, and includes hemodynamic and functional impairment, thrombo-embolic and bleeding events, and higher rates of renal failure.^29^

In 2017, Mojoli *et al.* considered 11 studies (11 033 patients) in their meta-analysis. Compared with sinus rhythm, long-term mortality was significantly higher in new-onset AF (OR 2.3, *P* < 0.0001) and preexisting AF groups (OR 2.8, *P* < 0.0001). Compared with sinus rhythm, preexisting AF increased the risk of late stroke (OR 1.3, *P* = 0.03), but not the risk of late bleeding.^30^

The interest in the study of the link between mortality and AF in the follow-up of TAVI patients could be seen also in more recent evaluation such as the one conducted by Baz *et al.* They proposed a score which included, in addition to biohumoral and functional parameters, AF as the only clinical characteristic, remarking on its prognostic value.^31^

Our data showed an increased risk of long-term mortality (until 5 years) in patients with AF and the present study represents a unique experience with long-term follow-up.

Regarding the hospitalization rate, our results indicated that patients with AF had a higher risk of hospitalization than SR patients, as demonstrated in the SOURCE-XT registry and in the FRANCE-2 study that evidenced an increased re-hospitalization rate due to heart failure.^11,32^ Despite all these observations, the AF status is not included in the available TAVI-specific risk scores yet.^33–35^ An additional factor which may contribute to high rates of hospital readmission and mortality in TAVI patients with AF is coexistent mitral regurgitation (MR). The interplay between MR, AF and mortality is complex and not fully understood in the TAVI setting. Preoperative MR (moderate or severe) is significantly more frequent in those with both preexisting and new-onset AF.^11,12^ On the other hand, the prevalence of AF increases together with increasing severity of MR. Multiple studies including two meta-analyses have shown that MR is associated with increased mortality after TAVI.^36–38^ Despite these inconsistencies, increasing evidence suggests that both MR and AF may have an independent influence on mortality in TAVI patients. Conversely, MR has not proved to be the only determinant of the high post-TAVI mortality rates in those with AF.^11,12^ Our findings show that AF at discharge influenced readmission, not only when assessed individually, but also when considered in the primary composite outcome (all-cause death and cardiovascular hospitalization) and the secondary composite outcome (all-cause death and all-cause hospitalization).

### Strengths and limitations

Our study is a real-world analysis of a multicentre, unselected population undergoing TAVI thus providing a unique and updated picture of these patients. One of the strengths of this analysis is the follow-up time which is longer than those of most previous studies. Additionally, most of the patients analysed had a low or intermediate STS score thus providing new insights into this population.

However, some limitations should be acknowledged. Our analysis has the intrinsic limitation typical of all observational and retrospective studies, linked to possible selection bias and the eventual presence of confounding factors. Our study did not include a comparison between preexisting and new-onset AF due to the low number of patients with new-onset AF in our database. The analysis also did not consider patients who may have developed AF throughout the entire follow-up and the types of AF during the follow-up are lacking. Another limitation is the absence of a more comprehensive evaluation of the clinical complexity of the patients and the evaluation of frailty. Finally, all patients were enrolled in tertiary centres of a single Italian region thus partly limiting the external validity of our results. For these reasons, our findings should be considered hypothesis-generating and further studies are warranted.

## Conclusions

Atrial fibrillation in patients treated with TAVI is associated with an increased risk of AKI, all-cause death, and hospitalization. It is uncertain if AF is a cause or a marker of sicker patients, but in any case, it is a factor that should be carefully evaluated for the patient's risk stratification. More studies are required to determine the best monitoring time range for this population.

## Acknowledgements

Funding: No funding was received for this work.

### Conflict of interest

G.B. received small speaker's fees from Boston, Boehringer, Bayer, Daiichi-Sankyo, Janssen and Sanofi, outside of the submitted work. The other authors declare no conflict of interest.
